# E-cigarette and hookah adoption patterns: Is the harm reduction theory just so much smoke?

**DOI:** 10.1016/j.abrep.2019.100246

**Published:** 2020-01-20

**Authors:** Eric W. Ford, Kitty S. Chan, Megha Parikh, Kevin B. Lowe, Timothy R. Huerta

**Affiliations:** a University of Alabama Birmingham, 1720 2nd Avenue South, Birmingham, AL 35294, United States; b MedStar Health Research Institute, Georgetown Surgical Outcomes Research, 6525 Belcrest Road, Suite 700, Hyattsville, MD 20782, United States; c Johns Hopkins Bloomberg School of Public Health, 624 N. Broadway, Baltimore, MD, 21205, United States; d The University of Sydney Business School, Australia; e Department of Family Medicine, The Ohio State University, Columbus, OH, United States

**Keywords:** Consumer behavior, Regulated products, Product innovation

## Abstract

**Aims:**

This paper explores inter-generational changes in consumers smoking product uptake and use patterns after the introduction of e-cigarettes and hookahs.

**Design:**

Item Response Theory (IRT) is used to analyze the Health Information National Trends Survey sponsored by the Food and Drug Administration (HINTS-FDA). The survey was fielded in 2015. IRT allows the pattern of product use to be described and help assess whether the new tobacco products (i.e., e-cigarettes, hookahs) serve as gateway to other products or act in harm reduction modality.

**Findings:**

The results indicate that the new product alternatives have changed the how tobacco products are adopted in the U.S. In particular, younger respondents were more likely to have engaged in cigar, e-cigarette and water-pipe use than the older cohort.

**Conclusions:**

The introduction of nicotine products previously unavailable in the U.S is creating new modes for smoking initiation in the age groups most likely to begin a new habit. There is little evidence that smokers in the older HINTS cohorts are using the e-cigarette as a smoking cessation tool. The rise of cigar use in the younger cohort may indicate that legal products are being mixed with illicit substances (i.e., ‘blunting’).

## Highlights

• Smoking initiation and progression patterns are changing with due to new products.
• E-cigarettes are potentially drawing new populations to nicotine addictive behavior.
• The role of cigars is changing. Flavored versions are different than prior generations’ products.

## 1 Introduction

Tobacco use is the largest cause of preventable disease and death in the U.S (Dietz, Douglas, & Brownson, 2016). Beginning with the Surgeon General’s 1964 warning on the hazards of smoking, there has been a steady increase in governmental efforts to reduce the availability of tobacco products and change consumers’ behavior (US Department of Health and Human Services, 2014). Interventions have included advertising restrictions, anti-smoking ads (e.g., the ‘truth’ campaign), increased taxation rates, public smoking bans, and lawsuits targeting tobacco companies for failing to disclose known hazards to consumers (Gruber, 2001, Thompson, 2005). The net effect has been an overall reduction in the number of habitual tobacco users and a reduced rate of those initiating the practice. Within this context, new and old technologies that deliver tobacco’s addictive chemical – nicotine – have been introduced into the U.S. marketplace. In particular, electronic nicotine delivery systems’ (ENDS) (i.e., e-cigarettes) represent a new technology. The water pipe (a.k.a., hookahs) is an older technology imported the Middle East that has migrated to the U.S. Both products use has grown rapidly since 2000 (Huerta, Walker, Mullen, Johnson, & Ford, 2016).

The introduction of smoking products into new populations where the potential harms and benefits are not well understood poses numerous public policy and marketing issues (Soneji, Sung, Primack, Pierce, & Sargent, 2018). In the case of smoking, product innovations and introductions that change consumers’ existing habit initiation pathways by sidestepping regulatory efforts may undermine public health goals (Moreau, Markman, & Lehmann, 2001). A significant concern with products like ENDS are that they may disrupt established use patterns by serving as a ‘hedonistic gateway’ to traditional cigarettes (Chartrand, 2005, Kandel, 1975, Keyes et al., 2016, Sutfin et al., 2015, Villanti et al., 2016). However, these same products may also offer a harm reduction strategy and cessation avenue for current smokers (Bolton et al., 2006, Payne, 2016). In cases where product introductions significantly alter consumer behaviors vis-à-vis other offerings, there is an increased need for information on both harms and benefits related to those products.

The purpose of this paper is to explore if new smoking initiation patterns are occurring in among U.S. consumers. Item response theory (IRT) is used to model younger and older cohorts’ experiences in consuming various smoking products (Oyserman, 2009). By modeling the likelihood of using a particular product given underlying level of engagement with tobacco use, IRT can be used to create a product “diffusion pathway” model that identifies the sequence various tobacco products are likely to be adopted by different age groups (DeBresson, 1991).

## 2 Background

Water pipes and ENDS represent two relatively new products in the U.S. marketplace and they are increasing in popularity among young consumers (Huerta et al., 2016). While the water pipe or hookah is an older technology imported into the U.S. from the Middle East and North Africa, it has seen a significant increase in its popularity in recent years. ENDS, on the other hand, are a new technology designed to circumvent smoking restrictions by removing tobacco from the experience and delivering the additive ingredient – nicotine – in a novel fashion. The American Lung Association reports that young adults (ages 18–24) are the consumers targeted by these products’ ads because they are three times more likely to initiate ENDS use than traditional cigarettes. Both products, hookah and ENDS gained their marketplace footholds without relying on traditional retail outlets (e.g., convenience stores). Instead, new specialized storefronts (e.g., Hookah bars and Vape shops) and Internet sellers were created to facilitate product distribution. As a social activity, water-pipe smoking typically takes place in dedicated ‘hookah bars’ or cafes that sell other smoking paraphernalia. In this sense, hookah use represents a group-driven form of hedonic pleasure. The group nature of the activity makes it more likely that younger consumers, who identify strongly with peers engaged in the smoking ritual will also partake despite the moral ambiguity of such consumption (Von Schuckmann, Barros, Dias, & Andrade, 2018).

ENDS products have been on the U.S. market since 2007. ENDS convert liquids (i.e., ‘juice’) containing nicotine into a vapor the consumer inhales (i.e., ‘vaping’). The liquid often contains flavor additives and other chemicals to make inhaling the nicotine component feel less caustic (Levy et al., 2017). Manufacturers have continued to innovate, introducing more compact technologies that are closer to the size and appearance of traditional cigars and cigarettes. The major tobacco companies entered the ENDS market in 2014 promoting the products as a safer alternative to cigarettes as a form of corporate social responsibility (Yoon, Gürhan-Canli, & Schwarz, 2006). The rapid growth in ENDS use is running counter to trends in cigarette, cigar and pipe use; all of which continued to drop (Pearson, Richardson, Niaura, Vallone, & Abrams, 2012).

With increased advertising and growing market share, ENDS are gaining in popularity among younger consumers (Padon, Maloney, & Cappella, 2017). Analysis shows that from 2009 to 2014, ENDS market share for all smoking products more than doubled annually. ENDS advertising is not banned from television or other media currently. Public health professionals have expressed concerns that advertising strategies that successfully increased smoking rates in the 1950s, 60s and 70s, augmented with newer social media campaigns, will promote ENDS use and create a new generation of smokers (Hawkins, Johnson, Denzel, Tercyak, & Mays, 2016). One of the primary ad messages is that ENDS carry no health risks.

ENDS manufacturers’ claim that their devices deliver lower-levels of harmful chemicals than regular cigarettes have been borne out in lab comparisons (Benowitz, Donny, & Hatsukami, 2017). The Centers for Disease Control and Prevention (CDC) is funding research to assess if switching to ENDS may be an effective method to assist in smoking cessation efforts. Two studies reported in 2011 showed that significantly more smokers reported a reduction or cessation of cigarette consumption at four months post-intervention by using ENDS, relative to a comparison group that did not use ENDS (Polosa et al., 2011, Siegel et al., 2011). However, other studies have found that smokers are less likely to quit when using ENDS (Grana, Benowitz, & Glantz, 2014). Hence, ENDS ads’ claims for their positive effect on smoking harm reduction or smoking cessation have not been supported consistently by empirical research.

### 2.1 Gateway theory of consumer behavior

A common concern among public health advocates is that consumers engaging in one risky behavior are likely to initiate other, still riskier activities. The classic ‘gateway’ pattern suggests that consumers initiate substance abuse behaviors by first using more readily available legal substances. For example, illicit drug users start by consuming alcohol (either legally or illegally obtained) and then migrate toward more harmful products such as smoking. Another version of the gateway model has consumers beginning with legally distributed products then transitioning to illicit substance use. For example, the U.S. heroin epidemic attributed to consumers migrating from prescription opioids to the illegal substance when the former became more closely regulated (Kolodny et al., 2015).

The social aspect of nicotine delivery is an important part of the gateway theory. Sequential progress from ‘milder’ to more ‘harmful’ substances depends heavily on the environment in which the exposure occurs (Aaron Ginzler, Cochran, Domenech-Rodríguez, Mari Cauce, & Whitbeck, 2003). Hookah use in particular has a peer-approval element as part of the communal smoking that leads users to underestimate potential health risks (Heinz et al., 2013). In addition, the ritual of assembling the hookah is akin to the IKEA effect where consumers feel more vested in the experience because they played an instrumental role (Norton, Mochon, & Ariely, 2012). Further still, hookah users report a greater willingness to engage in other risky behaviors (Berg et al., 2015, Heinz et al., 2013). There are concerns that ENDS use and hookah smoking may serve as an gateway behavior to traditional smoking paradigms (Fairchild, Bayer, & Colgrove, 2014). The novelty of the new devices may be particularly appealing to younger consumers (Manning, Bearden, & Madden, 1995). The research-to-date on gateway effects is most compelling with products that have both social activities associated with them and an addictive component such as alcohol or nicotine (Bretteville-Jensen, Melberg, & Jones, 2008). However, there is an alternative hypothesis that ENDS may serve to reduce the health-related harms associated with smoking cigarettes.

### 2.2 The tobacco harm reduction or gateway to better behaviors theory

One major aim of policymakers has been to get current cigarette smokers to quit the habit. The current standard of care is a set of pharmaceutical-based smoking cessation protocols that fail about 85% of the time even under optimal conditions (Moore et al., 2009, Pedersen et al., 2016). The flaws in the current “evidence-based” policies are they do not satisfy the consumers’ psychological desire to smoke, the treatment duration is too short, and there is no mechanism for self-reinforcement when the urge to smoke returns (Nitzkin, 2014). Hence the ability of current smoking cessation efforts to reduce harm is minimal at best. The introduction of ENDS presented those interested in mitigating the harm from habitual smoking with a new option.

None of the ‘smoking’ products available in the marketplace are risk free, but some have comparatively lower, long-term health impacts. Having cigarette smoker switch to ENDS is considered a desirable option for four reasons. First, ENDS do not have many of the carcinogenic agents found in traditional tobacco products. Next, ENDS do have the active ingredient (i.e., nicotine) self-dosed; therefore, they may be more effective satisfying psychological dependencies. Third, ENDS satisfy the consumers’ behavioral need to inhale that has become ingrained in many smokers. Fourth, the ENDS allows the social component associated with smoking habit to be continued. Hence, ENDS are considered to be a viable alternative for current smokers (Cahn & Siegel, 2011).

The two competing hypotheses can be thought of as a ‘gateway’ that swings in opposite directions. The traditional gateway opens onto a pathway that is a series of habits each more harmful than the last. Alternatively, the new nicotine product market entries may be a gateway that opens in a new direction allowing current smoker to switch paths onto one that is less harmful to their long-term health. It is possible to consider both gateways by looking at those entering the period of smoking initiation (i.e., younger consumers) and those whose likely initiation period has passed (i.e. older individuals). The study is designed to explore the two theories’ in two cohorts of the HINTS series.

## 3 Methods

Data for this analysis is drawn from the 2015 Health Information National Trends Survey-Food and Drug Administration (HINTS-FDA). HINTS is a NCI-sponsored nationally representative survey of Americans’ access and use of cancer-related health information. Analyses were conducted in accordance with NCI recommendations (Hesse, Moser, Rutten, & Kreps, 2006). The FDA version of the survey had questions designed (1) to assess tobacco-related communications received by current, former and never tobacco users; and (2) examine beliefs about tobacco products, tobacco product contents, and modified risk claims (Blake et al., 2016). The target population is the adult (aged ≥ 18) civilian non-institutionalized population of the United States. The HINTS-FDA was conducted from May through September 2015 by mail and uses a stratified two-stage sample design and oversamples current and former smokers. A total of 3738 respondents (33% response rate) completed the survey. Survey weights are used to account for the complex survey design and non-response.

For the analysis, age stratifications were collapsed into two groups to facilitate comparison of the ENDS innovations’ impact on older versus younger individuals’ product uptake patterns. While consumers are most likely to initiate a new smoking habit between 18 and 24; the 18–34 age group was created to have a sufficient sample size to measure the effects of ENDS introduction. The 35–49 sample represents smokers that initiated the habit prior to the innovations’ market introduction.

### 3.1 Measures

Seven items were employed to measure consumers’ engagement with smoking products. Six of the items were drawn from a question that asked respondents to indicate whether they had tried the following tobacco products even once: cigars, hookah or water pipe filled with tobacco, electronic cigarette, pipe filled with tobacco, “roll your own” cigarettes, or “snus”. Cigarette use was determined by asking respondents whether they had smoked at least 100 cigarettes in their entire lifetime. A total of 3723 (99.5%) respondents had positive response data on at least one of the survey items.

### 3.2 Statistical analysis

We used item response theory (IRT) modeling to examine the relationship of each indicator of tobacco use to the others (Maheswaran & Shavitt, 2000). IRT models draw upon information in the response patterns of a set of items used to measure a particular construct to estimate: (1) how strongly each item is related to the underlying construct (i.e., item discrimination, A) and (2) at what level of the construct the item measures best (i.e., item difficulty, B) (Cheng & Liu, 2012).

These properties, discrimination and difficulty, have analogues to our understanding associated with the diffusion of these substitutes. A higher item discrimination (A) parameter would indicate that use of a particular tobacco product is more strongly related to the overall engagement with tobacco products. In contrast, a higher item difficulty (B) parameter for a product would indicate a “harder” item, in that fewer respondents are likely to indicate having tried or used the product than for “easier” items. Given the positive correlations between items expected under the uni-dimensionality assumption, consumers at a particular level of engagement with smoking products use, would have a higher probability of having tried a smoking product with an “easier” difficulty parameter than a product with a “harder” location parameter (similar to greater likelihood of a test taker getting an easier math question right than a harder math question). This relationship between items allows for items to be ranked based on difficulty (B) parameter estimates. Such rankings of item difficulties can provide insight on the likelihood that a particular product has been used or tried as the engagement with tobacco products increases. This dynamic speaks directly to the gateway hypothesis – if we assume that populations adopt in accordance with the theorized sequential progress from ‘milder’ to more ‘harmful’ substances, then we should see this phenomenon manifest as an order of item difficulties, with lower difficulty estimates for milder substances and progressively higher difficulty parameters for increasing harmful substances. In this respect, then, we submit that the underlying methodological mechanism of IRT is well-suited to answer the gateway question.

A zero in item difficulty confidence interval should be interpreted as a tobacco product for which someone at the average tobacco engagement of the sample would have a 50% likelihood of having used the product. Thus, an item difficulty estimate of zero, or a confidence interval (CI) that includes zero, *should not* be interpreted as lacking statistical significance. Rather, it is whether or not CIs of adjacent items overlap that is the determining factor for significant differences in the items’ difficulty estimates. By evaluating the CIs of the item difficulty estimates, it is possible to assess if there are levels of the underlying construct of tobacco engagement that do not discriminate well or are measuring the same phenomenon. When sorted by *Item Difficulty* levels of the underlying construct where CIs do not overlap suggest that including an item in this gap would improve the differentiation of respondents along the construct. In practice, this would suggest a potential opening for a gateway technology to transition users from one product to another.

## 4 Results

Table 1 displays the samples’ demographics. The younger grouping (18–34 years of age) provided 455 respondents to the analysis that indicated some form of tobacco product use. The comparison sample (ages 35–49) had 3,162 survey participants who smoked in some form. Overall, cigarettes and cigars were the most commonly used substance across the entire sample (see Table 2). For the younger group of consumers, having ‘ever smoked a cigar’ was the most frequently acknowledged activity. For the older consumer cohort, having ‘ever smoked 100 or more cigarettes’ was the most common product use type.

**Table 1:** Survey sample demographics.

n = 3723	
Age, n(%)	
18–34	455 (29.7)
35–49	658 (24.4)
50–64	1222 (24.8)
65–74	756 (10.6)
75+	526 (7.9)
Male, n (%)	1495 (46.7)
Race/Ethnicity, n (%)	
Hispanic	241 (14.9)
Non-Hispanic White	2637 (59.8)
Non-Hispanic Black	231 (10.4)
Non-Hispanic Asian	119 (5.0)
Non-Hispanic Other	141 (2.1)
Educational Attainment, n (%)	
Less than High School	236 (10.9)
High School Graduate	724 (21.0)
Some College/Vocational school	1130 (32.9)
College Graduate or More	1572 (35.1)
Region	
Northeast	606 (18.1)
Midwest	1101 (21.2)
South	1382 (37.1)
West	634 (23.5)
Annual Household Income, n (%)	
Less than $20,000	661 (18.7)
$20,000 to <$35,000	504 (13.5)
$35,000 to <$50,000	413 (12.3)
$50,000 to <$75,000	605 (14.7)
$75,000 or more	1110 (32.4)
Any Health Insurance, n (%)	3434 (89.4)

*Missing data: Age (2.7%); Gender (5.0%); Race/Ethnicity (7.8%); Educational attainment (1.7%); Annual HH Income (8.4%); Health Insurance (2.3%).

**Weighted percentages

**Table 2:** Tobacco use.

	Total Population (n = 3723)	Ages 18–34 (n = 455)	Ages 35+ (n = 3162)
Cigarettes (≥100), No., (%)	1631 (39.8)	153 (30.9)	1435 (43.5)
Cigars, No., (%)	1300 (38.0)	218 (44.8)	1054 (35.2)
Hookah, No., (%)	384 (20.0)	168 (42.3)	209 (10.1)
Electronic Cigarette, No., (%)	497 (21.4)	138 (37.7)	351 (14.5)
Pipe filled with tobacco, No., (%)	624 (17.8)	64 (15.7)	545 (18.5)
Roll your own cigarettes, No., (%)	757 (24.9)	113 (27.3)	624 (23.6)
Snus, No., (%)	190 (9.9)	67 (18.1)	119 (6.2)

*Weighted percentages.

Factor analysis indicated that all of the smoking products behaved as a single construct and should be included in the IRT modeling. Collectively, the smoking products accounted for 84% of the variance (see Table 3). Table 4 displays the IRT results for the total sample and the two age groups. Overall, the *item Discrimination* parameters are greater than one for all questions indicating they provide adequate discrimination for differences in tobacco engagement. When comparing the two age groups, the younger sample had a different *Item Difficulty* ordering than the older group (see Table 4). The younger sample indicated that cigars, water pipes, cigarettes and ENDS were more likely to have been tried or used than traditional pipes, self-rolled cigarettes and snus. For the younger sample, cigars (*Item difficulty* = 0.085; CI = −0.046, 0.216) were the “easiest” substance to adopt and differ significantly from the other smoking forms because the confidence intervals do not overlap. The next three items, hookahs (*Item difficulty* = 0.471; CI = 0.276, 0.667), cigarettes (*Item difficulty* = 0.5155; CI = 0.378, 0.652) and ENDS (*Item difficulty* = 0.58; CI = 0.455, 0.716) had similar difficulty estimates and overlapping confidence intervals. The inference is that younger consumers view these three forms of smoking similarly and they are as likely to try or use one as the other – at least in the statistical sense.

**Table 3:** Factor analysis.

	Factor loading	Uniqueness
Cigarettes	0.765	0.189
Cigars	0.800	0.293
Hookah	0.591	0.392
Electronic Cigarette	0.733	0.235
Pipe filled with tobacco	0.752	0.221
Roll your own cigarettes	0.849	0.245
Snus	0.654	0.522

*Inter-item correlation range: 0.18–0.75.

**1 Eigen value >1; Explains 84% of the variance.

**Table 4:** IRT analysis.

	Total Population		Ages 18–34		Ages 35+	
	Item Discrimination	Item Difficulty (Rank)	Item Discrimination	Item Difficulty (Rank)	Item Discrimination	Item Difficulty (Rank)
Cigarettes	2.091	0.189 (0.136, 0.241)	2.917	0.515 (3) (0.378, 0.652)	2.192	0.133 (1) (0.077, 0.189)
Cigars	2.129	0.484 (0.428, 0.540)	2.814	0.085 (1) (−0.046, 0.216)	2.012	0.551 (2) (0.486, 0.616)
Roll your own cigarettes	3.579	0.864 (0.806, 0.921)	3.963	0.745 (5) (0.606, 0.883)	3.571	0.882 (3) (0.819, 0.946)
Pipe filled with tobacco	2.269	1.139 (1.061, 1.218)	2.680	1.262 (6) (1.045, 1.478)	2.403	1.089 (4) (1.007, 1.171)
Electronic Cigarette	1.721	1.489 (1.373, 1.605)	3.349	0.580 (4) (0.445, 0.716)	1.460	1.797 (5) (1.622, 1.973)
Hookah	1.198	2.111 (1.887, 2.335)	1.377	0.471 (2) (0.276, 0.667)	1.205	2.562 (6) (2.226, 2.898)
Snus	1.734 (1.459, 2.009)	2.270 (2.054, 2.486)	2.187	1.314 (7) (1.068, 1.559)	1.479	2.721 (7) (2.364, 3.079)

For the older respondents (ages 35–49), cigarettes had the lowest item difficulty (*Item difficulty* = 0.133; CI = 0.077, 0.189) indicating that it is the easiest to adopt. Cigarettes differ significantly from the next product, cigars, which were outside of the confidence interval (*Item difficulty* = 0.551; CI = 0.486, 0.616). The smoking patterns for this group had ENDS (*Item difficulty* = 1.797; CI = 1.622, 1.973), hookahs (*Item difficulty* = 2.562; CI = 2.226, 2.898) and snus (*Item difficulty* = 2.721; CI = 2.364, 3.079) with the highest difficulty estimates, and undifferentiated in their difficulty, suggesting that they are the least likely smoking activities to be taken up. Overall, these results indicate that for the older sample, cigarettes and cigars represent the first and easiest smoking pattern to adopt, with the transition to hookah and ENDS far more less likely. Older consumers were more likely simply to start with cigarettes than use a hookah or ENDS. In part, this may be because the innovative products were not available during their most likely smoking initiation ages. Moreover, it does not appear that the older cohort is using ENDS as harm reduction strategy substituting them for traditional products. Had this been the case, ENDS would have appeared adjacent to traditional cigarettes.

As a result, the younger and older samples differed significantly in their smoking preferences and patterns. In the younger cohort, hookah and ENDS served as comparable products to cigarette use. While the IRT does not demonstrate the clear gateway or diffusion pathway from one product to the next, it does indicate that the gateway may be wider than anticipated with ENDS and hookah serving as substitutes for cigarettes in the initiation phase. Hence, the products represent a group of smoking activities that are interchangeable for the younger consumers. For the older cohort, ENDS do not appear to be serving as a substitute for traditional tobacco product use. Taken together, it appears the ‘gateway’ theory is supported to some extent and the ‘harm reduction’ theory has to be rejected.

## 5 Discussion

The results support the hypothesis that recently introduced smoking alternatives are creating new gateways for young consumers to adopt the products (Leventhal et al., 2016). Both ENDS and water pipes were higher on the younger samples’ smoking percentage and item difficulty order than for their 35 and older counterparts (Cooper, Loukas, Case, Marti, & Perry, 2018). A clear implication is that younger smokers are more likely to use the innovative products than the older sample. In addition, the IRT analysis provides insights into the social circumstances that may contribute to initiation of various smoking habits. Specifically, hookah smoking may create a social environment where peers can reinforce positive perceptions of other products’ use including ENDS and the traditional cigarette (Agaku, Odani, Armour, & Glover-Kudon, 2018).

Hookah and ENDS products are often co-located in the retail outlets where the former is consumed by groups of smokers (Lee & Kim, 2015). As a result, social acceptance coupled with readily available access to smoking alternatives creates a consumer-retail environment where products can be used in an interchangeable fashion. In addition, the conditions involved in hookah smoking include introduction to the most widely available smoking product – cigarettes. Hence, the introduction of previously unavailable smoking products in the U.S. may lead to an increase in the uptake of traditional products among the age group most likely to initiate the habit. However, the paradigm may be more complicated than the information at hand allows for exploration.

Although ENDS were originally introduced as a safer alternative to cigarette use, there is increasing awareness that ENDS use does not discourage and may even encourage traditional cigarette use (Dutra & Glantz, 2014). The rise of cigars and water-pipes to the top of the younger age group’s use list may be linked to another topic *not* explored in the HINTS data – marijuana use. The 84% explanatory power of the factor analyses indicates there are other smoking products that may be in use or consumers know them by another name. The latter explanation, that there were products not included in the survey is the more likely as pictures of each smoking device accompanied the questions. Furthermore, both cigars and water pipes are used to facilitate marijuana smoking. Cigars are employed to reduce throat irritation, mask marijuana’s smell, and allow for public consumption (i.e., ‘blunting’) (Delnevo and Hrywna, 2006, Rosenberry et al., 2017). Similar to cigars, water-pipes can be used to consume marijuana and/or tobacco products – again with the reduced throat irritation. For poly-tobacco users, these delivery combinations with marijuana are among the most prevalent (Yu, Saddleson, Murphy, Giovino, & Mahoney, 2017). Therefore, it is logical that both cigars and water pipes would be part of the gateway to smoking habituation pathway.

## 6 Conclusion

The widespread availability of ENDS and water pipes are changing the smoking product adoption and use paradigm among the younger age groups that are most likely to initiate a new habit. Prior research findings have concluded that e-cigarette awareness and use are increasing over time (Huerta et al., 2016). Compared to older smokers, younger people are more likely to avail themselves of these product and use ENDS and / or water pipes. In addition, younger smokers appear to view water pipes, tobacco cigarettes and ENDS as being comparable. More concerning still, is the rise of ‘cigars’ to the top of the product usage list. This may indicate that illicit marijuana use is the new ‘gateway’ substance to other smoking product initiation.

The reverse gateway or harm reduction hypothesis that current consumers will substitute ENDS for traditional cigarettes was not supported. Older smokers are not as familiar with ENDS as the younger, comparison group. If traditional cigarette users were using ENDS as a substitute or cessation mechanism, the two products should have appeared adjacent to one another in the IRT ordering. While the results of this study are cross-sectional, the comparison of older and younger adopters’ smoking paradigms suggests a shift in both consumers’ uptake rates and the types of products used.

Collectively, the findings support the growing movement toward more regulation (e.g., product approval) and restrictions (e.g., limiting access) on smoking innovations, particularly those that target younger users such as ‘candy flavored’ options. While the harm reduction potential of substituting ENDS for traditional smoking products is desirable, it appears that innovative products may be serving to create more new smokers than would occur otherwise. Therefore, the overall potential harm to the population is likely to be increasing as initiation rates rise and effective substitution for current smokers’ lags.

## 7 Limitations

The sampling design attenuated the statistical power of the IRT analyses. Hookah and ENDS are innovations that would not have been available to those closer to the 34-year-old cutoff. These respondents may have grown-up under the older gateway paradigm where cigarettes served as the primary entry point product. In addition, the survey design did not include younger age groups that are the most vulnerable to social/peer pressures to initiate the smoking habit (e.g. 12–18 years of age). Collectively, the effect size of the *Item difficulty* scores may have been underestimated due to sampling frames. The pathway to smoking product use may be more pronounced than described herein with distinct step-by-step patterns representing multiple gateways appearing.

Another limitation of the study at-hand is the survey did not explore former smokers cessation experience. Ideally, information on the number of quit attempts, nicotine replacement support used, and the time since smoking would be assessed. Therefore, the study at-hand cannot adequately assess product abandonment and replacement fully.

## Role of funding sources

No external funding was used to conduct this study.

## Contributors

Authors Ford and Chan designed the study and wrote the protocol. Author Parikh conducted literature searches and provided statistical analyses. Authors Ford, Chan and Lowe drafted the Introduction, Methods and Results, and Discussion – respectively. Author Huerta wrote the Conclusions and Abstract. All authors contributed to and have approved the final manuscript.

## Declaration of Competing Interest

The authors have no conflict of interests to report.

## Appendix A Data collection information

The first author Health Information National Trends Survey is collected by National Institutes of Health as authorized by The Public Health Service Act, Section 411 (42 USC285 a). The OMB control number for the particular iteration of the survey used herein is OMB # 0925-0538. The authors jointly analyzed the data in accordance with the conditions provided by the National Institutes of Health when receiving the data (See below – https://hints.cancer.gov/).

In order for the Health Information National Trends Survey (HINTS) to provide a public-use or another version of data to you, it is necessary that you agree to the following provisions.

1. You will not present/publish data in which an individual can be identified. Publication of small cell sizes should be avoided.
2. You will not attempt to link nor permit others to link the data with individually identified records in another database.
3. You will not attempt to learn the identity of any person whose data are contained in the supplied file(s).
4. If the identity of any person is discovered inadvertently, then the following should be done;
  a. no use will be made of this knowledge,
  b. the HINTS Program staff will be notified of the incident,
  c. no one else will be informed of the discovered identity.
5. You will not release nor permit others to release the data in full or in part to any person except with the written approval of the HINTS Program staff.
6. If accessing the data from a centralized location on a time sharing computer system or LAN, you will not share your logon name and password with any other individuals. You will also not allow any other individuals to use your computer account after you have logged on with your logon name and password.
7. For all software provided by the HINTS Program, you will not copy, distribute, reverse engineer, profit from its sale or use, or incorporate it in any other software system.
8. The source of information should be cited in all publications. The appropriate citation is associated with the data file used. Please see Suggested Citations in the Download HINTS Data section of this Web site, or the Readme.txt associated with the ASCII text version of the HINTS data.
9. Analyses of large HINTS domains usually produce reliable estimates, but analyses of small domains may yield unreliable estimates, as indicated by their large variances. The analyst should pay particular attention to the standard error and coefficient of variation (relative standard error) for estimates of means, proportions, and totals, and the analyst should report these when writing up results. It is important that the analyst realizes that small sample sizes for particular analyses will tend to result in unstable estimates.
10. You may receive periodic e-mail updates from the HINTS administrators.
